# Current tobacco use is associated with higher rates of implant revision and deep infection after total hip or knee arthroplasty: a prospective cohort study

**DOI:** 10.1186/s12916-015-0523-0

**Published:** 2015-11-19

**Authors:** Jasvinder A. Singh, Cathy Schleck, W. Scott Harmsen, Adam K. Jacob, David O. Warner, David G. Lewallen

**Affiliations:** Medicine Service and Center for Surgical Medical Acute Care Research and Transitions, VA Medical Center, Faculty Office Tower, 510, 20th Street South, 805B, Birmingham, AL 35294 USA; Department of Medicine at School of Medicine and Division of Epidemiology at School of Public Health, University of Alabama, 1720 Second Ave. South, Birmingham, AL 35294-0022 USA; Department of Anesthesiology, Mayo Clinic College of Medicine, 200 1st St SW, Rochester, MN 55905 USA; Department of Biostatistics, Mayo Clinic College of Medicine, 200 1st St SW, Rochester, MN 55905 USA; Department of Orthopedic Surgery, Mayo Clinic College of Medicine, 200 1st St SW, Rochester, MN 55905 USA

**Keywords:** Arthroplasty, Complications, Outcomes, Smoker, Smoking, THA, THR, TKA, TKR, Tobacco use, Total hip replacement, Total knee replacement

## Abstract

**Background:**

Tobacco smoking is a risk factor for several adverse post-operative outcomes. We aimed to compare the rates of complications in current tobacco users and non-users who underwent primary total hip arthroplasty (THA) or total knee arthroplasty (TKA).

**Methods:**

All patients who underwent primary THA or TKA at the Mayo Clinic from 2010–2013 were included in the study. Current tobacco use was defined as the use of cigarettes, cigars, pipes, or smokeless tobacco reported at the time of index THA or TKA; current non-users were former users or never users. We used Cox proportional hazards regression to assess the association of current tobacco use status with each post-THA/TKA complication, using hazard ratios and 95 % confidence intervals (CI).

**Results:**

Tobacco use status was available for 7926 patients (95 %) and not available for 446 patients (5 %); 565 (7 %) were current tobacco users. Compared to non-users, current tobacco users  were more likely to be male (*p* < 0.001), and less likely to be obese (p ≤ 0.008), be older than 60 years, have Charlson score >0 or have undergone TKA rather than THA (p < 0.001 each). The hazard ratios for deep infection (2.37; 95 % CI 1.19, 4.72; *p* = 0.01) and implant revision (1.78; 95 % CI 1.01, 3.13; *p* = 0.04) were higher in current tobacco users than in non-users. No significant differences were noted for periprosthetic fractures or superficial infections.

**Conclusions:**

We noted that current tobacco use was associated with high risk of deep infection and implant revision after primary THA or TKA. Future studies should determine the optimal time for tobacco use cessation before elective surgeries such as THA and TKA to improve short-term and long-term arthroplasty outcomes.

## Background

Tobacco smoking is a risk factor for several adverse post-operative outcomes, including wound-related complications, in part owing to its effect on tissue oxygenation and inflammatory healing processes [1, 2]. For example, in a study of >33,000 patients undergoing total knee arthroplasty (THA) or total hip arthroplasty (TKA) using the Veterans Affairs Surgical Quality Improvement Program database, current smokers had higher risk of 30-day surgical site infections (SSIs) than non-smokers [3]. However, studies describing the association of smoking on short-term or long-term arthroplasty surgical outcomes are limited.

In a study that included 621 patients, the rate of arthroplasty revision was significantly higher in smokers compared to non-smokers in univariate analyses [4]; rates of other surgical complications were no different between the groups [4]. However, the analyses were not adjusted for other possibly important covariates such as age, gender, or comorbidity. In contrast, another study that examined the risk of implant revision in 1301 THA patients found no association between smoking and revision risk in ceramic-on-polyethylene bearing primary THA [5]. A systematic review of the effect of smoking on outcomes after total joint replacement showed that current smoking increased the risk of overall post-operative complications and death but that there were scarce data for smoking and surgical outcomes of arthroplasty [6]. To our knowledge, well-designed studies that have examined the risk of post-arthroplasty implant-related complications due to tobacco use are lacking. A recent meta-analysis of studies assessing the effect of smoking on THA outcomes acknowledged the lack of a consistent definition of “current smoker,” adjustment for important covariates, and heterogeneity among studies as key limitations [7].

The objective of this study was to assess the association between tobacco use status and outcomes of TKA or THA using data from an institutional Total Joint Registry. We hypothesized that current tobacco use would be associated with an increased risk of revision surgery, wound infections, and peri-prosthetic fractures after primary THA or TKA.

## Methods

We followed the Strengthening of Reporting in Observational studies in Epidemiology (STROBE) statement to describe this study and results [8]. The Institutional Review Board at Mayo Clinic, Rochester, MN, approved the study and waived the requirement for informed consent.

### Setting, participants, and data sources

This observational cohort study included all patients who underwent primary THA or TKA at Mayo Clinic from 2010 to 2013 and had tobacco use status documented in the nursing database. We obtained data from two sources, the Mayo Clinic Joint Registry and the nursing notes of the Mayo Clinic electronic medical record.

The Mayo Clinic Joint Registry is a prospective registry of all patients who undergo joint replacement surgery at the Mayo Clinic, Rochester, MN [9, 10]. Each patient who undergoes joint replacement surgery is followed prospectively with clinical follow-up at one, two, and five years, and every five years thereafter. Patients failing to return for a follow-up visit are sent a questionnaire (joint-specific) and asked to send in their radiographs. Those who fail to return the questionnaire are contacted on the telephone by trained registry staff. Patients undergo a brief telephone interview using a standardized questionnaire including complications, such as infection, fracture, and any additional surgery. Data, including the indication for surgery and operative findings, are requested for subsequent operations performed at other hospitals.

Using unique patient identifiers, we linked the tobacco use status data from the Mayo Clinic Anesthesia database to the Joint Registry. As a matter of routine clinical assessment, tobacco use is consistently documented at the time of admission for patients at all Mayo Clinic hospitals in Rochester, MN, by admission nursing staff. This includes whether patients are currently using tobacco, and the type of use (cigarette, cigar, pipe, or smokeless tobacco use).

### Predictor of interest

Current tobacco use status was the primary predictor of interest. Current tobacco users were defined as people who were using cigarettes, cigars, pipes, or smokeless tobacco at the time of their surgery, with the rest classified as non-users. Thus, current non-users included both never-users and former users.

### Covariates

We included several covariates known/suspected to be associated with complications after THA or TKA, namely demographics (age, gender); body mass index (BMI); American Society of Anesthesiologist (ASA) class; implant fixation (uncemented, cemented, antibiotic/vacuum); and medical comorbidity assessed using a validated Deyo–Charlson index [11], which is a weighted scale of 17 comorbidities (including cardiac, pulmonary, renal, hepatic disease, diabetes, cancer, HIV and so on), expressed as a summative score where a higher score indicates more comorbidity.

### Outcomes of interest

The a priori outcomes were deep infection, superficial infection, peri-prosthetic fracture, and the need for revision surgery. Exploratory outcomes included the reasons for revision (aseptic loosening, infection, and peri-prosthetic fracture). Deep infection was defined as infection below the fascia; other infections were categorized as superficial infections. Peri-prosthetic fracture was defined as the presence of proximal, distal, or both, or avulsion fracture. Revision surgery was defined as the occurrence of revision of one or more components of the THA or TKA.

### Sample size and potential bias

No formal sample size calculations were done, given the lack of previous studies providing effect size estimates. The Mayo Clinic Joint Registry includes every patient who has undergone THA or TKA at the institution. We anticipated that despite a large sample size, the rarity of these complications would limit the ability to perform multivariable-adjusted analyses. We decided a priori that where possible, we would adjust for the most important significant covariates and confounders, and where not possible, acknowledge this as a limitation. We selected a large enough sample by choosing all eligible patients from 2010 to 2013 to avoid a type II error. In very few cases of simultaneous (or sequential) bilateral THA or TKA in a patient, we used only one side (or the first procedure) to avoid correlated observations.

### Statistical analyses

Descriptive statistics were reported as number (percentage) or mean (standard deviation) as appropriate. Characteristics of patients with and without known tobacco use status were compared using logistic regression analyses, also done for current tobacco users versus non-users. Odds ratios and 95 % confidence intervals (CI) are presented. Cox proportional hazards regression was used to assess the association of tobacco use status with each outcome, reporting a hazard ratio (HR) and 95 % CI. Kaplan–Meier (KM) survival was used to estimate implant survival. The KM curves go to 25 months, because the number at risk drops below 10 patients at 25 months. A *p*-value of less than 0.05 was considered significant.

## Results

### Characteristics of patients with and without tobacco use status data

Tobacco use status was available for 7926 patients (95 %) and not available for 446 patients (5 %) undergoing primary THA or TKA. Compared to those without available tobacco use status assessment, patients with tobacco use data were more likely to be in the age groups 71–80 and >80 years (reference, age ≤60), and to be female; no significant differences were noted in BMI, implant fixation, Deyo–Charlson index score, or ASA class (Table 1).

**Table 1:** Comparison of characteristics of patients with and without current tobacco use assessment among patients who underwent primary THA or TKA

	Tobacco use assessed	Tobacco use not assessed	Odds Ratio	*p*-value
	(*n* = 7926)	(*n* = 446)	(95 % confidence interval)	
	N (%)	N (%)		
Knee	4,277 (94.5 %)	249 (5.5 %)	0.93 (0.76, 1.12)	0.44
Hip	3,649 (94.9 %)	197 (5.1 %)	1.0 (ref)	
Male	3516 (94.1)	219 (5.9)	0.82 (0.68, 0.99)	0.04
Female	4410 (95.1)	227 (4.9)	1.0 (ref) ^a^	
Age ≤60	2430 (93.8)	161 (6.2)	1.0 (ref) ^a^	
61–70	2522 (94.2)	154 (5.8)	1.10 (0.88, 1.39)	0.40
71–80	2226 (95.5)	104 (4.5)	1.44 (1.12, 1.86)	0.005
>80	748 (96.5)	27 (3.5)	1.86 (1.22, 2.81)	0.004
BMI < 25	1177 (95.4)	57 (4.6)	1.0 (ref) ^a^	
25–29	2418 (94.4)	144 (5.6)	0.82 (0.60, 1.12)	0.21
30–34	2169 (95.2)	109 (4.8)	0.97 (0.70, 1.35)	0.86
35–39	1235 (94.4)	73 (5.6)	0.83 (0.58, 1.18)	0.30
≥40	889 (93.8)	59 (6.2)	0.74 (0.50, 1.08)	0.12
Cemented	2309 (94.6)	133 (5.4)	1.12 (0.71, 1.76)	0.62
Antibiotic/vacuum	2502 (94.6)	142 (5.4)	1.12 (0.74, 1.71)	0.59
No cement	3115 (94.8)	171 (5.2)	1.0 (ref) ^a^	
Charlson Index = 0	3734 (94.6)	212 (5.4)	1.0 (ref) ^a^	
>0	4192 (94.7)	234 (5.3)	1.02 (0.84, 1.24)	0.81
ASA = 1	231 (95.4)	11 (4.6)	1.0 (ref) ^a^	
2	5455 (94.8)	298 (5.2)	0.89 (0.48, 1.64)	0.70
3	2195 (94.3)	133 (5.7)	0.80 (0.43, 1.51)	0.49
4	42 (91.3)	4 (8.7)	0.51 (0.15, 1.66)	0.26

*ASA* American Society of Anesthesiologist, *BMI* body mass index in kg/m^2^, *Ref* reference category, *SD* standard deviation

^a^ the model was adjusted for the joint type (TKA vs. THA)

### Cohort characteristics

Of the 7926 patients, 565 (7 %) reported current tobacco use, with 7361 (93 %) not currently using tobacco (including never-users and past tobacco users). In the group of 565 current tobacco users, only two patients were classified as using smokeless tobacco only. In unadjusted analyses, compared to current tobacco non-users, current tobacco users were more likely to be male (*p* < 0.001), and less likely to be obese (p ≤ 0.008), older than 60 years, have Charlson score >0 or have undergone TKA rather than THA (p < 0.001 each). (Table 2). There were no significant differences in implant type or ASA class (Table 2). The mean follow-up was similar between current tobacco user and current tobacco non-users (349 versus 308 days, respectively).

**Table 2:** Cohort characteristics of THA/TKA patients with and without current tobacco use

	Current tobacco user	Past tobacco user or never used tobacco	Odds ratio	*p*-value
	(*n* = 565)	(*n* = 7361)	(95 % confidence interval)	
	N (%)	N (%)		
Knee	228 (5.0 %)	4049 (95.0 %)	0.55 (0.46, 0.66)	<0.001
Hip	337 (8.8 %)	3312 (91.2 %)	1.0 (ref)	
Male	326 (9.3)	3190 (90.7)	1.71 (1.44, 2.04)	<0.001
Female	239 (5.4)	4171 (94.6)	1.0 (ref) ^a^	
Age ≤60	354 (14.6)	2076 (85.4)	1.0 (ref) ^a^	
61–70	143 (5.7)	2379 (94.3)	0.38 (0.30, 0.46)	<0.001
71–80	56 (2.5)	2170 (97.5)	0.16 (0.12, 0.22)	<0.001
>80	12 (1.6)	736 (98.4)	0.10 (0.06, 0.18)	<0.001
BMI <25	118 (10.0)	1059 (90.0)	1.0 (ref) ^a^	
25–29	157 (6.5)	2261 (93.5)	0.66 (0.51, 0.85)	0.001
30–34	144 (6.6)	2025 (93.4)	0.70 (0.54, 0.91)	0.008
35–39	90 (7.3)	1145 (92.7)	0.82 (0.61, 1.09)	0.17
≥40	51 (5.7)	838 (94.3)	0.65 (0.46, 0.92)	0.02
Implant fixation				
Cemented	124 (5.4)	2185 (94.6)	0.90 (0.62, 1.29)	0.57
Antibiotic/vacuum	151 (6.0)	2351 (94.0)	0.96 (0.69, 1.32)	0.81
No cement	290 (9.3)	2825 (90.7)	1.0 (ref) ^a^	
Charlson Index = 0	309 (8.3)	3425 (91.7)	1.0 (ref) ^a^	
>0	256 (6.1)	3936 (93.9)	0.76 (0.63, 0.90)	0.001
ASA class 1	18 (7.8)	213 (92.2)	1.0 (ref) ^a^	
2	393 (7.2)	5062 (92.8)	1.05 (0.64, 1.72)	0.85
3	148 (6.7)	2047 (93.3)	1.00 (0.60, 1.68)	0.99
4	5 (11.9)	37 (88.1)	1.72 (0.60, 4.95)	0.31

*ASA* American Society of Anesthesiologist, *BMI* body mass index in kg/m^2^, *Ref* Reference category, *SD* standard deviation

^a^ the model was adjusted for the joint type (TKA vs. THA)

### Current tobacco use and the risk of post-THA/TKA complications

**Fig. 1:**
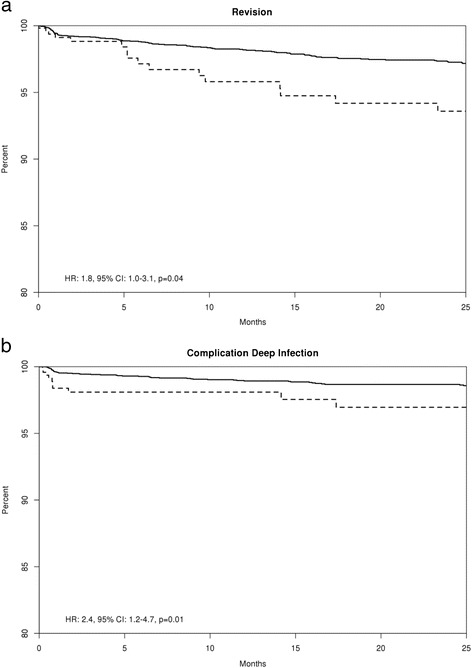
**a** Survival free of implant revision for any reason and **b** survival free of deep infection in current tobacco users (*dashed line*) compared with current tobacco non-users (*solid line*). *CI* confidence interval, *HR* hazard ratio

In analyses that adjusted for joint type (THA versus TKA), age, and sex, current tobacco users had significantly higher hazard of deep infection, (HR 2.37; 95 % CI 1.19, 4.72; *p* = 0.01) and implant revision (HR 1.78; 95 % CI 1.01, 3.13; *p* = 0.04) than current tobacco non-users (Table 3). No significant differences in periprosthetic fractures and superficial infection were noted (Table 3). The times free of implant revision and free of deep infection by current tobacco use are shown in Fig. 1a and b, respectively. In exploratory analyses, in a fully adjusted model for joint type (THA versus TKA), age, and sex, current users tended to be at higher risk of revision for infection (HR 2.28; 95 % CI 0.99, 5.27; *p* = 0.05). Other exploratory outcomes occurred at much lower frequencies that did not allow meaningful analyses to be conducted, that is, revision owing to aseptic loosening occurred only in 14 patients and revision owing to peri-prosthetic fracture in only nine patients.

**Table 3:** Adjusted for joint type and multivariable-adjusted hazard of each complication by current tobacco use status

Endpoint	Current tobacco user	Number of events	1 year (95 % CI)	2 years (95 % CI)	HR (95 % CI)^a^	*p*-value	HR (95 % CI)^b^	*p*-value
Any revision	Yes	16	95.8 (93.4, 98.3)	93.6 (90.5, 96.8)	**1.98 (1.14, 3.46)**	**0.02**	**1.78 (1.01, 3.13)**	**0.04**
	No	111	98.2 (97.8, 98.6)	97.3 (96.7, 97.8)	1.0 (ref)		1.0 (ref)	
Deep infection	Yes	10	98.1 (96.8, 99.4)	97.0 (94.9, 99.0)	**2.45 (1.25, 4.79)**	**0.009**	**2.37 (1.19, 4.72)**	**0.01**
	No	57	98.9 (98.6, 99.2)	98.7 (98.3, 99.0)	1.0 (ref)		1.0 (ref)	
Superficial infection	Yes	4	99.0 (98.1, 100)	99.0 (98.1, 100)	1.06 (0.38, 2.98)	0.92	1.07 (0.37, 3.09)	0.90
	No	50	99.1 (98.8, 99.3)	99.1 (98.8, 99.3)	1.0 (ref)		1.0 (ref)	
Peri-prosthetic fracture	Yes	10	98.2 (97.0, 99.4)	98.2 (97.0, 99.4)	0.84 (0.44, 1.58)	0.59	1.01 (0.53, 1.92)	0.97
	No	134	98.0 (97.6, 98.3)	97.7 (97.3, 98.2)	1.0 (ref)		1.0 (ref)	

^a^Cox models adjusted for knee (versus hip); ^b^Cox models adjusted for knee (versus hip), male (versus female), and age at surgery (per 1 year). Significant hazard ratios and *p*-values are in bold

## Discussion

Smoking can interfere with wound healing and increase infection risk by multiple potential mechanisms, including vasoconstriction, which can interfere with wound healing [12]; fibroblast survival and migration at the site of healing [13]; and carbon monoxide from smoking can lead to decreased oxygen delivery to tissues [14, 15], and oxygen is needed for optimal wound healing [16]. In this study of 7926 primary THA or TKA patients, we found that current tobacco use was associated with a significantly higher risk of implant revision and deep infection. These differences remained significant in multivariable-adjusted models that included joint type, age, and gender.

Several prior studies have examined the effect of smoking status on infectious complications in patients undergoing total joint arthroplasty. In a study of 3908 patients with orthopedic implants, including THA and TKA or fracture internal fixation devices, smoking was a risk factor for SSIs for up to one year after surgery [17]. In our previous large study of 33,336 veterans who underwent THA or TKA, current smokers had an adjusted odds ratio of 1.41 for 30-day SSIs compared to never smokers [3]. In the current study, we found that current tobacco users were at 2.37-times higher odds of developing post-surgical deep infection compared to current non-users. Our previous study [3] was performed in veterans, who are sicker than the general US population [18], but the current study cohort is more representative of THA/TKA cohorts. Studies that examined a related, but somewhat differently defined outcome, that is, wound or local complications, reported contradictory results—one study found smoking was associated with wound complications with odds of 2.15 [19], while another found no difference in local complications between smokers and non-smokers (3.8 % versus 3.8 %) [20]. A systematic review of smoking and outcomes after total joint replacement showed that current smoking increased the risk of any post-operative complication and death [6]. Another systematic review that included any surgery showed that current smoking was associated with SSI and wound complications with odds ratios of 1.79 (95 % 1.57–2.04) and 2.27 (95 % 1.82–2.84) [2].

Our finding that current smokers were younger and had lower medical comorbidity than current non-smokers is similar to the younger age and lower ASA class in current smokers undergoing THA or TKA compared to their counterparts in the Veterans Affairs study of >33,000 patients [3].

Our study extends these studies of infectious complications by studying all-comers who underwent primary THA or TKA at the Mayo Clinic, using a large sample, examining superficial and deep infections separately, and performing multivariable-adjusted analyses. Even though we had a large sample size, the frequency of some complications was low, which may have led to a type II error, that is, lack of power, potentially explaining some negative findings. An interesting observation was that, even when non-significant, odds ratios for several post-operative complications ranged from 1.5 to 2.0 in current tobacco users.

The most novel finding in our study was that current tobacco users had 1.8-fold higher hazard of the risk of revision arthroplasty than non-users, with 117 cases of implant revision in a cohort of 7926 patients. Our finding in univariate association was confirmed in multivariable-adjusted analyses, suggesting that the finding was robust. Our finding is in contrast to few previous studies [5, 21, 22] and agrees with one recent study [4] showing the association of smoking and revision risk. Espehaug et al. studied 1628 THA patients in a matched case–control study from the Norwegian Arthroplasty Register and found that current smokers had similar odds of 0.8 (95 % CI 0.5, 1.3) for reoperation as the non-smokers [21]; study limitations were that smoking status was assessed at the time of survey post-arthroplasty and the definition of “early revision” was unclear. No association between smoking and revision risk was found in 1301 patients (34 revisions) with ceramic-on-polyethylene bearing primary THA, with odds ratio of 1.3 (95 % CI 0.6, 2.5) [5]. Meldrum et al. retrospectively studied 147 patients with THA performed by a single surgeon and found that in multivariable models adjusted for age, gender, BMI, diagnosis, stem fixation, and alcohol use, smokers had 4.5-times the hazard of revision than non-smokers, a non-significant finding (*p* = 0.07) [22]. A single-center study of 621 TKA patients with 18 revisions at mean follow-up of 4 years (131 current smokers with 11 revisions) showed that the risk of revision in smokers was significantly higher than in non-smokers [4]. Most previous studies of revision risk had small sample sizes leading to potential type II error, that is, missing a significant association when one is present, owing to an underpowered study. Even with <60 revisions due to infection, the HR related to current smoking almost reached significance, 2.28 (95 % CI 0.99, 5.27; *p* = 0.05). This correlates well with the biology and the proposed pathophysiology of impaired healing due to tobacco-related toxic effects on fibroblasts and other cells needed for optimal wound healing [12–16].

Our study included a large sample from a Joint Registry, with systematic data collection and monitoring for complications. The higher overall revision risk with current tobacco use may be mediated via a higher rate of associated deep infections in the early post-operative period; the borderline significance of rates of revision for infection further supports this hypothesis.

Our study had several limitations. Findings may not be generalizable to other settings, because this was a single-center study. However, the similarity of our THA/TKA cohorts to other published studies of THA/TKA [23, 24] as well as the US Nationwide Inpatient Sample [25], supports generalizability. Owing to the cohort study design, our study is subject to residual confounding. Confounding due to unmeasured factors among patients undergoing THA/TKA compared to those who are denied the surgery owing to higher medical comorbidity needs to be considered while interpreting study findings. Despite a large sample size, most outcomes of interest were uncommon. Therefore, we likely missed some important associations owing to type II errors, that is, missing an important observation when one existed because of small sample size. Larger sample sizes are needed to definitively answer these questions. Pack-year smoking history was not available for the majority of patients and therefore could not be analyzed.

The number of patients only using smokeless tobacco was extremely small (2 of the 565 current smokers), therefore, no subgroup analyses could be performed by the type of tobacco use. As with all observational studies, these findings need to be reproduced in other joint registries of large size (state or country-based registries, Kaiser Permanente etc.) that prospectively assess smoking status and post-arthroplasty complications. Selection bias needs to be considered while interpreting these findings, because patients undergo pre-operative assessment before having THA/TKA and it is possible that those patients who underwent THA/TKA are healthier than the age-matched and sex-matched general population not undergoing these procedures, or patients who undergo less invasive procedures. These findings should not be generalized to the general population or those populations undergoing less invasive procedures.

## Conclusion

We confirmed that current smokers are at increased risk for infectious complications after total joint arthroplasty. In addition, we now show that current smokers are also at risk for adverse functional outcomes that require revision arthroplasty. It is well established that tobacco use interventions reduce perioperative risks in a variety of surgeries (including orthopedic surgery) [26, 27]. Further studies would be needed to determine if tobacco use intervention specifically can reduce the risk of deep infections and revision surgery. However, because revision is a relatively infrequent event, such studies would need to be large. It would be more practical to have SSI or wound complications as the primary outcome and revision as the secondary outcome. Given the multiple benefits of smoking cessation to both short-term and long-term health, and that most primary TKA and THA are elective, our findings provide further impetus for the routine application of effective tobacco use interventions to all current smokers scheduled for these procedures.
